# Efficacy and safety of ferric citrate tablets in Chinese patients with hyperphosphatemia undergoing maintenance hemodialysis: a multicenter, randomized, open-label, active-controlled, phase III trial

**DOI:** 10.1080/0886022X.2025.2534019

**Published:** 2026-08-31

**Authors:** Jie Ma, Bicheng Liu, Jialiang Pang, Pei Wang, Ying Li, Caili Wang, Donghui Zheng, Xiaoping Wang, Xiumei Hu, Jianrong Zhao, Xinzhou Zhang, Changchun Cao, Zunsong Wang, Li Xing, Suhua Li, Hong Cheng, Jian Huang, Yi Yang, Xiaolan Chen, Kun Ye, Tiekun Yan, Lianhua Chen, Guojuan Zhang, Feng Zheng, Siyuan Teng, Qing Zhu, Hong Liu, Xuemei Li

**Affiliations:** ^a^ Department of Nephrology, Peking Union Medical College Hospital, Chinese Academy of Medical Sciences and Peking Union Medical College, Beijing, China; ^b^ Department of Nephrology, Zhongda Hospital, Southeast University, Nanjing, China; ^c^ Blood Purification Center, Xinxiang First People’s Hospital, Xinxiang, China; ^d^ Blood Purification Center, The First Affiliated Hospital of Zhengzhou University, Zhengzhou, China; ^e^ Department of Nephrology, The Third Hospital of Hebei Medical University, Shijiazhuang, China; ^f^ Department of Nephrology, The Frist Affiliated Hospital Baotou Medical College, Baotou, China; ^g^ Department of Nephrology, Huai’an Second People’s Hospital, Huai’an, China; ^h^ Department of Nephrology, Blood Purification Center, Central Hospital Affiliated to Shandong First Medical University, Jinan, China; ^i^ Department of Nephrology, Zhuzhou Central Hospital/Zhuzhou Hospital, Affiliated to Xiangya School of Medicine, Central South University, Zhuzhou, China; ^j^ Department of Nephrology, The Affiliated Hospital of Inner Mongolia Medical University, Hohhot, China; ^k^ Department of Nephrology, Shenzhen People’s Hospital, The Second Clinical Medical College of Jinan University, Shenzhen, China; ^l^ Department of Nephrology, Sir Run Run Hospital Nanjing Medical University, Nanjing, China; ^m^ Department of Nephrology, The First Affiliated Hospital of Shandong First Medical University, Shandong Provincial Qianfoshan Hospital, Jinan, China; ^n^ Hemodialysis Room, People’s Hospital of Zhengzhou, Zhengzhou, China; ^o^ Nephrology Center, The First Affiliated Hospital of Xinjiang Medical University, Urumqi, China; ^p^ Department of Nephrology, Beijing Anzhen Hospital, Capital Medical University, Beijing, China; ^q^ Department of Nephrology, Jinhua Municipal Central Hospital, Jinhua, China; ^r^ Department of Nephrology, The Fourth Affiliated Hospital, Zhejiang University School of Medicine, Yiwu, China; ^s^ Department of Nephrology, Affiliated Hospital of Nantong University, Nantong, China; ^t^ Department of Nephrology, The People’s Hospital of Guangxi Zhuang Autonomous Region, Guangxi Academy of Medical Sciences, Nanning, China; ^u^ Department of Nephrology, Tianjin Medical University General Hospital, Tianjin, China; ^v^ Department of Nephrology, Huai’an First People’s Hospital, Huai’an, China; ^w^ Department of Nephrology, Beijing Tongren Hospital, Capital Medical University, Beijing, China; ^x^ Department of Nephrology, The Second Affiliated Hospital of Dalian, Medical University, Dalian, China; ^y^ Department of Nephrology, Henan Provincial People’s Hospital, Zhengzhou, China; ^z^ Department of Nephrology, the Second Xiangya Hospital of Central South University, Changsha, China

**Keywords:** Chronic kidney disease, hyperphosphatemia, hemodialysis, ferric citrate, iron-based phosphorus binder

## Abstract

**Background:**

This study aimed to evaluate the efficacy and safety of ferric citrate tablets in Chinese patients with hyperphosphatemia undergoing maintenance hemodialysis (MHD).

**Methods:**

In this phase III, multicenter, randomized, open-label, non-inferiority trial, patients with hyperphosphatemia on maintenance hemodialysis were randomly assigned to receive either ferric citrate or sevelamer carbonate tablets for 12 weeks. The primary endpoint was the change in serum phosphorus levels from baseline to week 12, with a non-inferiority margin of 0.32 mmol/L. Secondary endpoints included changes in serum calcium, intact parathyroid hormone, and safety assessments.

**Results:**

A total of 239 patients were randomized to the ferric citrate group (*n* = 119) or the sevelamer carbonate group (*n* = 120). The mean change in serum phosphorus levels was −0.70 ± 0.50 mmol/L in the ferric citrate group and −0.61 ± 0.59 mmol/L in the sevelamer carbonate group (least squares mean difference, −0.09 mmol/L; 95% CI, −0.24 to 0.05 mmol/L; non-inferiority margin, 0.32 mmol/L). No significant inter-group differences were found in the percentage of patients achieving target phosphorus levels (49.09% vs. 48.28%, *p* = 0.902). Ferric citrate significantly improved iron-related parameters and hemoglobin levels. Most treatment-emergent adverse events were mild, with gastrointestinal disorders being the most common.

**Conclusions:**

Ferric citrate tablets were non-inferior to sevelamer carbonate in reducing serum phosphorus levels in hyperphosphatemia patients on maintenance hemodialysis, with the added benefit of improving iron-related anemia and a favorable safety profile.

## Introduction

In a recent cross-sectional study, the prevalence of chronic kidney disease (CKD) among Chinese adults was found to be 8.2% [1]. Based on the 6th national population census, approximately 82 million adults in China are living with CKD. The number of dialysis patients in China has rapidly increased from 255.11 per million population (PMP) in 2013 to 419.39 PMP in 2017, with projections estimating it will reach 629.67 PMP by 2025 [2]. Hyperphosphatemia is one of the most common complications in patients undergoing maintenance hemodialysis (MHD), significantly contributing to disorders of calcium-phosphorus metabolism and secondary hyperparathyroidism. In CKD patients, hyperphosphatemia acts as a key factor initiating metastatic calcification in cardiac valves, blood vessels, and soft tissues. It is also an independent risk factor for the progression of CKD, as well as cardiovascular morbidity and mortality [3–5]. A meta-analysis of 12 studies revealed that for every 1 mg/dL (0.323 mmol/L) increase in serum phosphorus levels, the risks of kidney failure and all-cause mortality rose by approximately 36% and 20%, respectively [4]. The newly published China Dialysis Calcification Study (CDCS) reported that the prevalence of vascular calcification among patients on maintenance hemodialysis in China was as high as 78.8%, with hyperphosphatemia identified as a significant risk factor for the progression of coronary artery calcification [6,7].

The management strategy for hyperphosphatemia includes dietary phosphorus restriction, adequate dialysis, and the appropriate use of phosphorus binders. Currently available oral phosphorus binders include calcium-based binders (e.g., calcium acetate, calcium carbonate) and non-calcium-based binders (e.g., sevelamer, lanthanum carbonate). However, calcium-based phosphorus binders may increase the risk of hypercalcemia and vascular calcification, while newer non-calcium-based binders are associated with gastrointestinal side effects [8,9]. Consequently, there remains a significant unmet need for safe and effective phosphorus binders.

Ferric citrate, an iron-based oral phosphorus binder, binds dietary phosphorus in the intestine to form insoluble ferric phosphorus, which is then excreted in feces, thereby reducing intestinal phosphorus absorption and lowering serum phosphorus levels. Additionally, ferric citrate offers the added benefit of iron repletion. Ferric citrate tablets have been approved for the treatment of hyperphosphatemia in CKD patients on dialysis and for iron deficiency anemia in those not on dialysis in both the United States and Japan. However, due to differences in dietary phosphorus intake, baseline iron status, and the prevalence of gastrointestinal disorders, the clinical response to ferric citrate may differ across populations. Therefore, this study aims to evaluate the efficacy and safety of ferric citrate tablets in Chinese CKD patients undergoing MHD with hyperphosphatemia.

## Methods

### Study design

This was a phase III, multicenter, randomized, open-label, active-controlled (sevelamer carbonate), 12-week trial. The trial was conducted in accordance with the Declaration of Helsinki and the National Medical Products Administration (NMPA) guidelines for Good Clinical Practice (GCP). This study has been approved by the ethics committees at the participating institutions. The study was registered at ClinicalTrials.gov (NCT04456803) and the China Drug Trials Registration and Information Disclosure Platform (CTR20190978).

### Patients

The inclusion criteria for this study required participants to be between 18 and 75 years old, undergoing MHD (at least 3 times per week in the 3 months before the inclusion) with a stable treatment regimen for CKD-MBD-related drugs for more than 1 month with unchanged dosage. Participants needed to have a serum phosphorus level between 1.97 and 3.23 mmol/L after washout, a Kt/Vurea ≥1.2 or URR ≥65%, and an expected survival greater than 6 months. Key exclusion criteria included high serum ferritin (≥800 ng/mL), TSAT levels (≥50%), blood transfusions with 3 months or hemoglobin ≤60 g/L, intact parathyroid hormone (iPTH) >1000 pg/mL, severe gastrointestinal or liver diseases, recent cerebrovascular or cardiovascular events, uncontrolled diabetes or hypertension, pregnancy, active malignancy, and participation in other clinical trials within 1 month. To reduce assessment bias, laboratory tests were conducted by blinded independent personnel. The eligibility criteria were detailed in Supplementary 1. Written informed consent has been obtained from all participants.

### Treatments

This study included a 2-week washout period followed by a 12-week treatment period. During the washout period, all previously administered phosphorus binders were discontinued. Patients who remained eligible after the washout period were 1:1 randomized to receive either ferric citrate tablets or sevelamer carbonate tablets (active comparator) for 12 weeks.

Ferric citrate tablets (Sinomune Pharmaceutical Co., Ltd) were administered orally three times daily (TID) immediately after each meal. The initial dose was 1500 mg/day (250 mg ferric citrate anhydrous per tablet) for 2 weeks, with adjustments up to 6000 mg/day (8 tablets TID) based on serum phosphorus levels measured at weeks 2, 4, 6, and 8. The dose was increased by 2 tablets per dose if serum phosphorus exceeded 1.78 mmol/L, decreased by 1 tablet per dose if serum phosphorus dropped below 1.13 mmol/L, and remained unchanged if serum phosphorus was between 1.13-1.78 mmol/L. Sevelamer carbonate tablets (manufactured by Genzyme Ireland Limited) were also administered orally TID with each meal. The starting dose was determined by serum phosphorus level on day 0, with 1 tablet per dose (800 mg/tablet) for serum phosphorus levels of 1.78–2.42 mmol/L and 2 tablets per dose for serum phosphorus levels ≥2.42 mmol/L. From week 3 to week 12, the dose of sevelamer carbonate was adjusted based on serum phosphorus levels measured at weeks 2, 4, 6, and 8, up to a maximum of 15 tablets/day (5 tablets TID). The dose adjustment plan is presented in Supplement 2.

During the study, concomitant use of drugs with phosphorus-binding properties (e.g., aluminum-, magnesium-, lanthanum-, or calcium-containing drugs) and other drugs that may affect phosphorus absorption was prohibited. However, intravenous iron preparations were allowed when deemed necessary by the investigator to treat renal anemia. When corrected serum calcium concentrations fell below 2.1 mmol/L, calcium supplements were permitted at the investigator’s discretion. Treatments for comorbidities, such as hypertension and diabetes, were allowed throughout the study. Symptomatic treatments were permitted for adverse events (e.g., diarrhea or constipation) that occurred during the study. Dose adjustments of ferric citrate or sevelamer carbonate tablets were also allowed in cases of severe gastrointestinal intolerance. Patients were advised to maintain a low-phosphorus diet, and dialysis prescriptions remained constant throughout the trial.

### Efficacy assessments

The primary endpoint of this study was the change in serum phosphorus levels at the end of the treatment (week 12) compared to baseline. Secondary endpoints included changes in serum phosphorus levels from baseline to weeks 2, 4, 6, and 8; the area under the curve of serum phosphorus levels over time from week 0 to 12 (AUC0-12); the proportion of subjects achieving target serum phosphorus levels (defined as serum phosphorus ≤1.78 mmol/L and ≥1.13 mmol/L) at weeks 4, 6, 8, and 12; changes in corrected serum calcium levels from baseline to weeks 4, 8, and 12; and changes in iPTH levels from baseline to weeks 4, 8, and 12.

### Safety assessments

Safety assessments included vital signs, physical examinations, laboratory tests (e.g., hematological tests, liver and kidney function, serum lipids, electrolytes), physiological tests (e.g., standard 12-lead electrocardiograms), and measurements of serum ferritin, serum iron, transferrin saturation (TSAT), and their changes from baseline to weeks 4, 8, and 12. All adverse events (AEs) and serious adverse events (SAEs) were recorded throughout the study duration.

### Detection

Serum biomarkers including phosphorus, calcium, albumin, and iron-related parameters were measured using standardized laboratory procedures across all participating centers. Specifically, serum phosphorus and calcium levels were determined using the phosphomolybdate method and o-cresolphthalein complexone method, respectively, with the AU680 analyzer (Beckman Coulter Inc.), which was upgraded to the AU5800 model on 29 March 2020. The intact parathyroid hormone (iPTH) levels were assessed *via* electrochemiluminescence immunoassay (ECLIA) using the ROCHE E601 analyzer. Serum albumin and transferrin saturation (TSAT) were measured using the bromocresol green method and iron saturation calculation method on the AU5800 analyzer, respectively. The detection of ferritin and other iron-related parameters was performed using the ARCHITECT i2000SR analyzer (Abbott Diagnostics) employing chemiluminescent microparticle immunoassay (CMIA) techniques. All samples were processed from serum specimens collected at specified study visits (i.e., visits 1, 2, 3, 4, 5, 6, and 7) to ensure consistency in monitoring and evaluation across the study period.

### Statistical analyses

Non-inferiority would be established if the upper limit of the 95% confidence interval (CI) for the mean treatment difference in serum phosphorus levels was below the non-inferiority margin (0.32 mmol/L). Following the intention-to-treat (ITT) principle, the full analysis set (FAS) included all participants who were randomly assigned, received at least one dose of study medication, and had at least one post-treatment efficacy evaluation. The per-protocol set (PPS) included participants who received all study medication as per the study design, without significant protocol violations, and completed all evaluations. All participants who were randomly assigned, received at least one dose of study medication, and had at least one safety evaluation, were included in the safety set (SS), which served as the primary population for safety assessment.

Statistical analyses were conducted using the SAS system, version 9.4. For all analyses, two-sided *p*-values ≤0.05 were considered statistically significant unless otherwise specified. Quantitative data were described using mean, standard deviation, median, minimum, and maximum values. Between-group comparisons of changes were made using t-tests or Wilcoxon rank-sum tests, depending on the characteristics of the variables. Categorical data were described using frequencies and percentages, with between-group comparisons conducted using chi-square or Fisher’s exact tests as appropriate. The Cochran-Mantel-Haenszel (CMH) chi-square test was employed for comparing between-group changes in stratified data.

## Results

### Baseline characteristics

**Figure 1.**
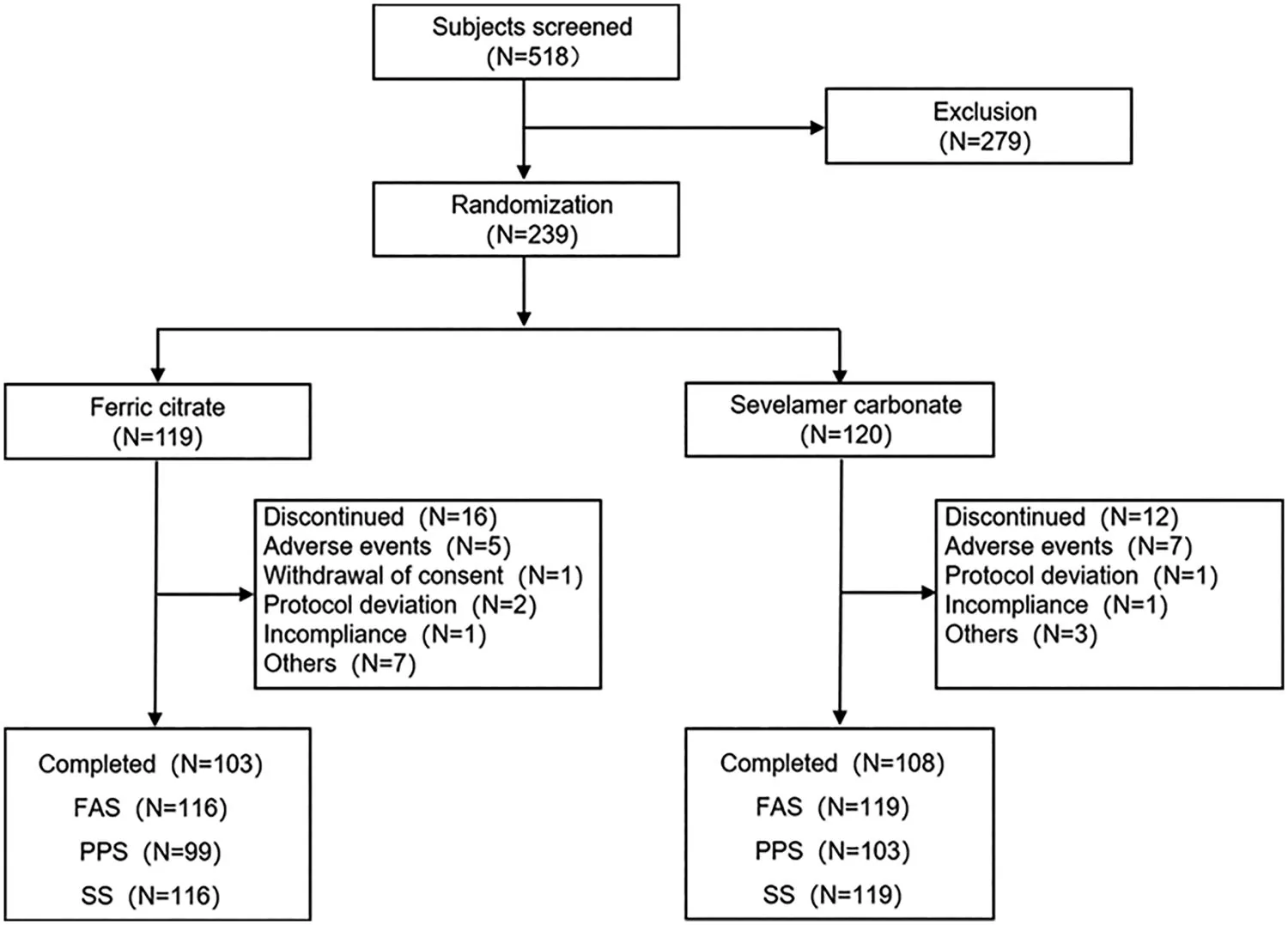
Study flow chart.

From May 2019 to September 2022, A total of 239 patients across 28 hospitals in China were enrolled in this study and randomized in a 1:1 ratio to receive either ferric citrate tablets (*n* = 119) or sevelamer carbonate tablets (*n* = 120). Of these, 235 patients were included in the full analysis set (FAS) population, and 202 patients were included in the per-protocol set (PPS) population (Figure 1). No significant differences were observed in baseline demographic characteristics or laboratory parameters between the two groups (Table 1).

**Table 1.** Demographic and baseline characteristics (FAS analysis population).

	Ferric Citrate (*n* = 116)	Sevelamer Carbonate (*n* = 119)	*p* Value
Sex, *n* (%)			
Male	56 (48.28%)	63 (52.94%)	0.474
Female	60 (51.72%)	56 (47.06%)	
Age (years)			
Mean±*SD*	50.65 ± 11.05	48.01 ± 11.57	0.113
BMI (kg/m^2^)			
Mean±*SD*	23.34 ± 3.31	23.00 ± 3.67	0.385
Parameters (Mean±*SD*)			
Serum phosphate (mmol/L)	2.42 ± 0.42	2.37 ± 0.47	0.406
Corrected serum calcium (mmol/L)	2.23 ± 0.28	2.26 ± 0.24	0.374
Intact PTH (pg/mL)	373.17 ± 216.07	338.04 ± 221.82	0.135
Serum ferritin (ng/ml)	189.59 ± 190.74	186.49 ± 186.27	0.981
Serum iron (μmol/L)	13.21 ± 4.49	12.88 ± 3.89	0.544
Transferrin saturation (%)	29.73 ± 10.61	28.36 ± 8.82	0.283
Hemoglobin (g/L)	114.14 ± 15.68	116.84 ± 15.37	0.235

### Serum phosphorus levels

The changes in serum phosphorus levels after treatment were assessed in both the FAS and PPS populations. In the FAS, the change in serum phosphorus level from baseline to week 12 was −0.70 ± 0.50 mmol/L in the ferric citrate group and −0.61 ± 0.59 mmol/L in the sevelamer carbonate group (least squares mean difference: −0.09 mmol/L; 95% CI: −0.24 to 0.05 mmol/L) (Table 2). In the PPS, the change was −0.77 ± 0.48 mmol/L in the ferric citrate group and −0.62 ± 0.59 mmol/L in the sevelamer carbonate group (least squares mean difference: −0.15 mmol/L; 95% CI: −0.29 to 0.00 mmol/L). The upper limit of the 95% CI for the difference did not exceed the non-inferiority margin of 0.32 mmol/L in both the FAS and PPS.

**Table 2.** Changes in laboratory parameters (mean ± *SD*, FAS).

	Visits (week)	Ferric Citrate (*n* = 116)	Sevelamer Carbonate (*n* = 119)	*p* Value
Changes in serum phosphorus level from baseline (mmol/L)	2	−0.33 ± 0.56	−0.44 ± 0.54	0.125
	4	−0.58 ± 0.47	−0.57 ± 0.57	0.938
	6	−0.63 ± 0.46	−0.64 ± 0.53	0.921
	8	−0.73 ± 0.54	−0.61 ± 0.56	0.221
	12	−0.70 ± 0.50	−0.61 ± 0.59	0.187
Serum phosphorus (AUC_0–12_)	0-12	22.54 ± 4.24	22.12 ± 3.90	0.479
Changes in corrected serum Calcium from baseline (mmol/L)	4	0.04 ± 0.23	0.02 ± 0.28	0.972
	8	0.07 ± 0.26	0.05 ± 0.24	0.549
	12	0.11 ± 0.24	0.02 ± 0.28	0.026
Changes in iPTH from baseline (pmol/L)	4	−42.80 ± 124.20	−11.64 ± 141.19	0.169
	8	−52.47 ± 176.65	−38.23 ± 146.66	0.418
	12	−49.41 ± 177.39	−15.13 ± 152.85	0.067

**Figure 2.**
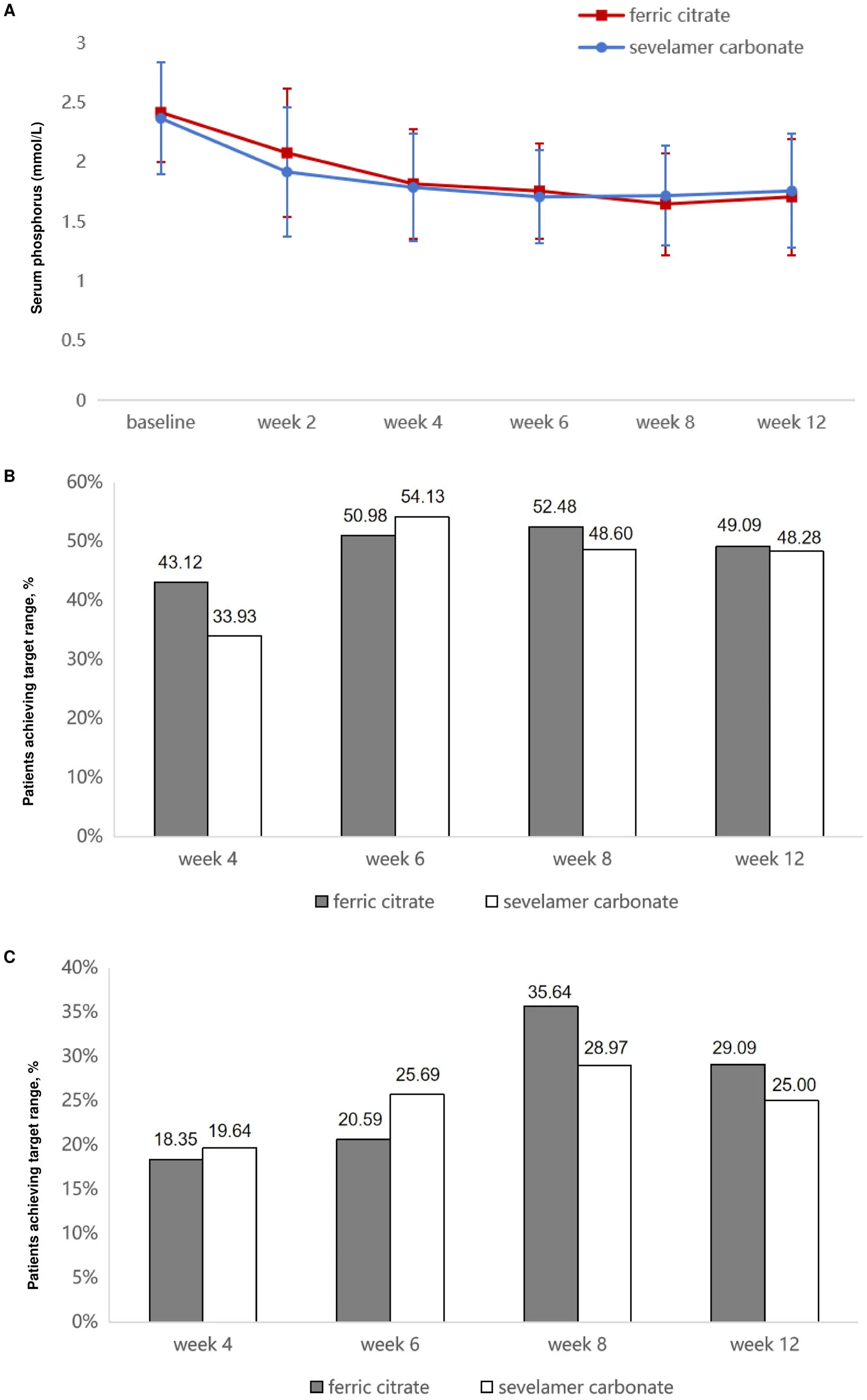
Changes of serum phosphorus levels. (A) Changes in serum phosphate over time (mean±*SD*, FAS); (B) Percentage of patients with serum phosphorus 1.13–1.78 mmol/L (FAS); (C) Percentage of patients with serum phosphorus ≤1.45 mmol/L (FAS).

At weeks 2, 4, 6, and 8, serum phosphorus levels were significantly reduced from baseline in both the ferric citrate and sevelamer carbonate groups (*p* < 0.0001). In the ferric citrate group, serum phosphorus levels significantly declined at week 2 (2.08 ± 0.54 mmol/L vs. baseline 2.42 ± 0.42 mmol/L, *p* < 0.001), further decreasing to 1.76 ± 0.40 mmol/L at week 6 and 1.71 ± 0.49 mmol/L at week 12 (Figure 2A).

There were no significant differences between the two groups in the percentage of patients achieving target serum phosphorus levels (1.13–1.78 mmol/L) (49.09% vs. 48.28% at week 12, *p* = 0.902) or ≤1.45 mmol/L (29.09% vs. 25.00% at week 12, *p* = 0.489) at any visit (Figure 2B and 2C). Additionally, there were no significant differences in serum phosphorus AUC0-12 between the two groups (Table 2).

### Serum calcium and iPTH

**Figure 3.**
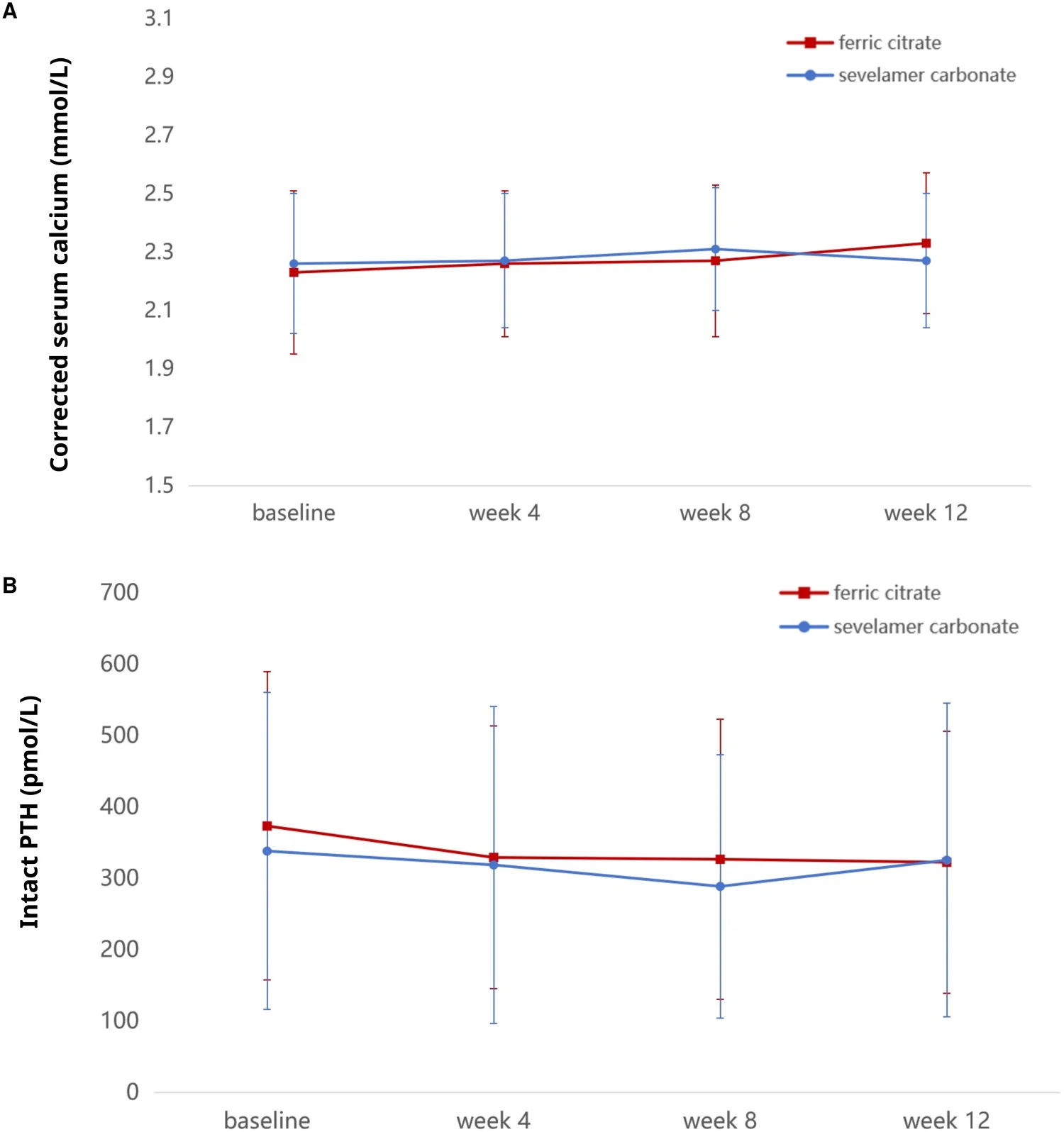
Changes of serum calcium and iPTH. (A) The absolute values of the corrected serum calcium at each visit (mean ± SD, FAS); (B) The absolute values of the iPTH at each visit (mean±*SD*, FAS).

The changes in corrected serum calcium from baseline to the end of treatment were 0.11 ± 0.24 mmol/L in the ferric citrate group and 0.02 ± 0.28 mmol/L in the sevelamer carbonate group (*p* = 0.026) (Table 2) (Supplementary 5). iPTH levels significantly decreased from baseline at weeks 4, 8, and 12 in the ferric citrate group (*p* = 0.001, 0.001, and 0.002, respectively). However, there was no significant difference between the two groups in the change in iPTH at week 12 (*p* = 0.067) (Figure 3).

### Safety

A total of 235 treated patients were included in the safety analysis set, with 90/116 (77.59%) in the ferric citrate group and 92/119 (77.31%) in the sevelamer carbonate group experiencing at least one TEAE. Most of these events were mild. One death occurred in the sevelamer group due to cardiac failure, which was considered unrelated to sevelamer. A single case of bleeding peptic ulcers, possibly related to the treatment, was reported as a SAE in the ferric citrate group. Gastrointestinal disorders were the most commonly observed adverse events by system organ class (SOC), including diarrhea (8.62% vs. 0.84%), constipation (3.45% vs. 6.72%), nausea (3.45% vs. 6.72%), vomiting (1.72% vs. 7.56%), discoloration of feces (9.48% vs. 0.00%), gastroesophageal reflux disease (0.86% vs. 6.72%), and gastrointestinal disorder (5.17% vs. 0.84%) in the ferric citrate and sevelamer carbonate groups, respectively (Table 3).

**Table 3.** TEAE During treatment.

	Ferric Citrate (*n* = 116) *N* (%)	Sevelamer (*n* = 119) *N* (%)
Any TEAE	90 (77.59)	92 (77.31)
Any serious TEAE	12 (10.34)	10 (8.4)
Any TEAE leading to study discontinuation	20 (17.24)	26 (21.85)
Any TEAE leading to death	0 (0)	1 (0.84)
Any treatment-related TEAE	53 (45.69)	35 (29.41)
Any serious treatment-related TEAE	1 (0.86)	0 (0)
Any treatment-related TEAE leading to study discontinuation	4 (3.45)	7 (5.88)
Any treatment-related TEAE leading to death	0 (0)	0 (0)
Most common treatment-related TEAEs (incidence ≥ 5%)		
Diarrhea	10 (8.62)	1 (0.84)
Constipation	4 (3.45)	8 (6.72)
Nausea	4 (3.45)	8 (6.72)
Vomiting	2 (1.72)	9 (7.56)
Discoloration of feces	11 (9.48)	0 (0)
Gastroesophageal reflux disease	1 (0.86)	8 (6.72)
Gastrointestinal disorder	6 (5.17)	1 (0.84)

### Iron-related parameters and hemoglobin

At weeks 4, 8, and 12, serum iron, ferritin, TSAT, and hemoglobin levels significantly increased from baseline in the ferric citrate group, while no significant changes were observed in the sevelamer carbonate group (Table 4). In the ferric citrate group, 4 (3.45%) patients experienced abnormal serum iron levels, 4 (3.45%) experienced abnormal TSAT levels, and 2 (1.72%) experienced abnormal serum ferritin levels.

**Table 4.** Changes in iron-related parameters and hemoglobin (mean ± *SD*, safety analysis set).

	Visits	Ferric Citrate (*n* = 116)	Sevelamer Carbonate (*n* = 119)	*p* Value
Serum iron (μmol/L)	Baseline	13.21 ± 4.49	12.88 ± 3.89	0.544
	Week 12	16.68 ± 7.43	13.89 ± 6.29	0.001
Serum ferritin (ng/mL)	Baseline	189.59 ± 190.74	186.49 ± 186.27	0.981
	Week 12	271.40 ± 219.14	199.72 ± 190.71	0.002
Transferrin saturation (%)	Baseline	29.73 ± 10.61	28.36 ± 8.82	0.283
	Week 12	40.50 ± 16.58	29.27 ± 15.04	<0.001
Hemoglobin (g/L)	Baseline	114.14 ± 15.68	116.84 ± 15.37	0.235
	Week 12	120.33 ± 18.89	113.09 ± 15.06	0.0015

## Discussion

Hyperphosphatemia is a common complication in patients on maintenance hemodialysis, predisposing them to an elevated risk of calcium-phosphorus metabolism disorders, secondary hyperparathyroidism, and cardiovascular morbidity. It is also an independent risk factor for CKD progression and all-cause mortality. In the present study, no statistically significant inter-group differences were observed in the relative changes in serum phosphorus from baseline or in serum phosphorus AUC_0-12_, demonstrating the non-inferiority of ferric citrate tablets compared to sevelamer carbonate tablets. The changes in corrected serum calcium and iPTH from baseline were also similar between the two groups, consistent with findings from previous studies in Japanese patients [10]. Data from a 52-week open-label study further confirmed that long-term treatment with ferric citrate tablets can maintain corrected serum calcium and iPTH levels in hemodialysis patients [11]. We hypothesized that treatment response in Chinese patients might differ due to distinct baseline dietary and anemia profiles, and our results confirmed consistent efficacy with added anemia benefits.

Controlling hyperphosphatemia has emerged as a key component in the management of CKD patients on hemodialysis. However, the optimal serum phosphorus levels in CKD patients remain controversial. The 2011 KDOQI guideline suggests maintaining phosphorus levels between 1.13 and 1.78 mmol/L. In the DOPPS5 study, serum phosphorus levels in Chinese patients were poorly controlled, with only 41.5% of patients achieving the target range (1.13–1.78 mmol/L). In this study, nearly 50% of patients in both groups achieved the target serum phosphorus levels at weeks 6, 8, and 12, consistent with previous studies in Japanese patients [10].

Given the serious impact of hyperphosphatemia on CKD prognosis, current clinical practice guidelines, such as the 2017 KDIGO guideline and the 2019 Chinese guideline, recommend lowering phosphorus levels toward the normal range (0.87–1.45 mmol/L) [8,9]. In this study, the rate of serum phosphorus levels below 1.45 mmol/L at week 12 showed no significant difference between the two groups, aligning with findings from a questionnaire survey covering 21 provinces in China (51,039 hemodialysis patients) and a real-world survey in Sichuan province (7053 hemodialysis patients), where only 26.7% of patients achieved target values below 1.45 mmol/L [12,13].

Renal anemia is another critical issue in patients with advanced CKD. Data from the Chinese cohort in the DOPPS study indicated that the average hemoglobin (Hb) level was 107 g/L in Chinese hemodialysis patients, with 18.8% having Hb levels <90 g/L, and only 34.4% reaching the target Hb level [12,13]. Iron absorbed *via* ferric citrate tablets could contribute to the treatment of renal anemia. Pharmacoeconomic studies in Japan and the United States have shown that ferric citrate tablets are more cost-effective than other phosphorus binders [14,15]. In this study, the ferric citrate group showed a significant increase in Hb levels from baseline to the end of the treatment compared to the sevelamer group. While other iron-based phosphorus binders, such as sucroferric oxyhydroxide, have demonstrated effective phosphorus control, studies have shown only modest increases in hemoglobin levels, which are less significant compared to those achieved with ferric citrate [16,17]. Additionally, sucroferric oxyhydroxide tablets are chewable but have been reported to be sticky, which could negatively impact patient compliance. Furthermore, the prescribing information for sucroferric oxyhydroxide lists tooth discoloration as the gastrointestinal disorder [18]. In contrast, the ferric citrate tablets used in our study did not require chewing, potentially providing greater convenience for patients.

Selecting appropriate phosphorus binders require careful consideration of the risk-benefit profiles of the drugs, along with the concurrent calcium-phosphorus metabolism disorders in patients. A systematic review and network meta-analysis of 12 phosphorus-lowering drugs indicated that ferric citrate tablets have substantial advantages in primary outcomes and are relatively safe in terms of drug-related serious adverse events [19]. In current study, ferric citrate tablets demonstrated a favorable safety profile in hemodialysis patients. Previous studies on oral ferric citrate identified gastrointestinal disorders, such as diarrhea, constipation, nausea, vomiting, and fecal discoloration, as the most common adverse events. No additional safety signals were observed in the present study. Although gastrointestinal AEs were mostly mild, they may influence long-term compliance. Patient counseling, gradual dose escalation, and symptomatic management could mitigate these effects in clinical practice.

Iron overload is a major concern in iron-based treatments. In this study, 4 (3.45%) patients in the ferric citrate group experienced abnormal serum iron levels, 4 (3.45%) had abnormal TSAT levels, and 2 (1.72%) had abnormal serum ferritin levels, all of which were considered mild to moderate. Serum ferritin and TSAT levels significantly increased from baseline in ferric citrate-treated patients, but all iron-related tests at each visit did not exceed the target levels recommended by clinical guidelines (serum ferritin <800 μg/L, TSAT <50%) [20,21]. No TEAEs or changes in liver function indicators were attributed to iron overload in this study. The safety of oral ferric citrate tablets in dialysis patients was also demonstrated in a post-marketing surveillance study involving 2,723 CKD patients, including both dialysis and non-dialysis patients [22]. In that long-term observational study, iron-related tests gradually increased after the initiation of ferric citrate treatment and stabilized after week 36, with serum ferritin in most patients not exceeding the recommended target level up to week 104. We recommend periodic monitoring of ferritin and TSAT levels during extended use, with dose reduction if levels approach the upper safety limits.

One limitation of this study is its open-label design, which may introduce potential bias. Nevertheless, this risk was mitigated by the use of objective laboratory-based endpoints and centralized assessments conducted by blinded evaluators. Further long-term investigations are needed to confirm the sustained efficacy and safety of ferric citrate, with particular attention to iron metabolism and cardiovascular outcomes.

In conclusion, current study indicate that ferric citrate tablets effectively reduce serum phosphorus levels in CKD patients receiving regular hemodialysis, with a favorable tolerability and safety profile. The phosphorus-lowering effect of ferric citrate tablets was non-inferior to sevelamer carbonate tablets, while the anemia-improving effect was significantly superior to that of sevelamer carbonate tablets. Most clinical adverse events and laboratory abnormalities were mild and well tolerated by the patients.

## Supplementary Material

Supplements.docx

## Data Availability

The data that support the findings of this study are not publicly available due to their containing information that could compromise the privacy of research participants. However, they are available from the corresponding author, Xuemei Li, upon reasonable request. Please contact lixmpumch@126.com for further inquiries.
