# Supplementary material for: Efficacy and safety of ferric citrate tablets in Chinese patients with hyperphosphatemia undergoing maintenance hemodialysis: a multicenter, randomized, open-label, active-controlled, phase III trial
Source: Ren Fail. 2026 Aug 31;48(1):2534019. doi: 10.1080/0886022X.2025.2534019 (PMC13537050; doi:10.1080/0886022X.2025.2534019)
Supplement: Supplementary file 1 [file IRNF_A_2534019_SM1271.docx]

**Supplement 1** Eligibility criteria

Inclusion Criteria:

1. Between the age of 18 and 75 years; no gender limitation;
2. Patients who maintain the hemodialysis schedule (including hemofiltration (HF) hemodialysis (HDF) hemoperfusion (HP)) as not less than 3 times a week in the 3 months before random enrollment.
3. Patients with a serum phosphorus level between 1.97 to 3.23 mmol/L (excluding the boundary value) after washout.
4. Kt/Vurea ≥1.2 or URR ≥65%.
5. Before the screening period, CKD-MBD related drug treatment is stable for more than one month, including the use of vitamin D (active vitamin D, vitamin D analogues, etc.) or calcimimetics (cinacalcet, etc.) and the dose remains unchanged.
6. The expected survival is greater than 6 months.
7. Willing to give written informed consent.

Exclusion Criteria:

1. Patients with a serum ferritin level ≥800 ng/mL or TSAT ≥50%.
2. Patients with hemochromatosis or patients receiving treatment for iron overload, or patients with paroxysmal sleep hemoglobinuria.
3. Patients who received blood transfusions within 3 months prior to Screening, or patients with hemoglobin ≤60 g/L.
4. Patients with intact-PTH >1000 pg/mL
5. Patients complicated with any of the following gastrointestinal diseases: acute peptic ulcer, chronic ulcerative colitis, localized enteritis, intestinal obstruction, habitual constipation (number of stools once per week), and chronic diarrhea (number of stools four times per day), or patients with a history of gastrectomy or enterectomy or patients who had undergone gastrointestinal surgery within 3 months prior to Screening, or patients with dysphagia.
6. Patients with impaired liver function (hepatic dysfunction or serum total bilirubin, aspartate aminotransferase or alanine aminotransferase ≥ 2 times the upper limit of normal) or patients with cirrhosis.
7. Patients with a history of parathyroidectomy (PTx) or percutaneous anhydrous ethanol injection (PEIT) within 6 months.
8. Patients with uncontrolled diabetes or uncontrolled high blood pressure or current active infectious diseases such as active viral hepatitis.
9. Patients with a history of severe allergies may be allergic to research drugs.
10. Patients with cerebrovascular disease (cerebral infarction, cerebral hemorrhage, etc.) or cardiovascular disease (congestive heart failure of Class III or severer in NYHA classification) requiring hospitalization within 6 months prior to Screening, or patients who use antiarrhythmic drugs to control arrhythmias or who use antiepileptic drugs to control seizures.
11. Patients who plan to receive a kidney transplant during the study period.
12. Patients with a history of drug and alcohol abuse
13. Patients with active or advanced malignancy.
14. Women who are pregnant or lactating
15. Patients complicated with active bleeding or requiring anticoagulation therapy with citrate in hemodialysis
16. Patients who had participated in other clinical studies within 1 month prior to Screening.
17. Patients who are not suitable for participating in the trial according to the investigator's judgment

**Supplement 2** Dosages adjustment plan

| Starting dose | | |
| --- | --- | --- |
| **Ferric Citrate:** 2 tablet per dose, 3 times/day | | |
| **Sevelamer Carbonate:** 1 tablet per dose (800mg/tablet) if serum phosphorus was 1.78-2.42 mmol/L, 2 tablets/dose if serum phosphorus was ≥2.42 mmol/L, 3 times/day | | |
|  | | |
| The dosage is adjusted according to serum phosphorus level as below | | |
| Serum Phosphorus (mmol/L) | Adjustment of Ferric citrate tablets | Adjustment of Sevelamer carbonate tablets |
| > 1.78 | Increase: 2 tablets, thrice daily | Increase: 1 tablet, thrice daily |
| 1.13–1.78 | No adjustment | No adjustment |
| < 1.13 | Decrease: 1 tablet, thrice daily | Decrease: 1 tablet, thrice daily |
| Note: Up to | 24 tablet per day | 15 tablet per day |
